# Multivalent vaccines demonstrate immunogenicity and protect against *Coxiella burnetii* aerosol challenge

**DOI:** 10.3389/fimmu.2023.1192821

**Published:** 2023-07-18

**Authors:** Sharon Jan, Alycia P. Fratzke, Jiin Felgner, Jenny E. Hernandez-Davies, Li Liang, Rie Nakajima, Algimantas Jasinskas, Medalyn Supnet, Aarti Jain, Philip L. Felgner, D. Huw Davies, Anthony E. Gregory

**Affiliations:** ^1^Vaccine Research & Development Center, Department of Physiology & Biophysics, University of California, Irvine, Irvine, CA, United States; ^2^Department of Microbial Pathogenesis and Immunology, Texas A&M Health Science Center, Bryan, TX, United States; ^3^Department of Pathology, Charles River Laboratories, Reno, NV, United States

**Keywords:** multivalency, *Coxiella burnetii*, subunit vaccine, adjuvant, aerosol challenge, guinea pig, hypersensitivity, reactogenicity

## Abstract

Vaccines are among the most cost-effective public health measures for controlling infectious diseases. *Coxiella burnetii* is the etiological agent of Q fever, a disease with a wide clinical spectrum that ranges from mild symptoms, such as fever and fatigue, to more severe disease, such as pneumonia and endocarditis. The formalin-inactivated whole-cell vaccine Q-VAX^®^ contains hundreds of antigens and confers lifelong protection in humans, but prior sensitization from infection or vaccination can result in deleterious reactogenic responses to vaccination. Consequently, there is great interest in developing non-reactogenic alternatives based on adjuvanted recombinant proteins. In this study, we aimed to develop a multivalent vaccine that conferred protection with reduced reactogenicity. We hypothesized that a multivalent vaccine consisting of multiple antigens would be more immunogenic and protective than a monovalent vaccine owing to the large number of potential protective antigens in the *C. burnetii* proteome. To address this, we identified immunogenic T and B cell antigens, and selected proteins were purified to evaluate with a combination adjuvant (IVAX-1), with or without *C. burnetii* lipopolysaccharide (LPS) in immunogenicity studies *in vivo* in mice and in a Hartley guinea pig intratracheal aerosol challenge model using *C. burnetii* strain NMI RSA 493. The data showed that multivalent vaccines are more immunogenic than monovalent vaccines and more closely emulate the protection achieved by Q-VAX. Although six antigens were the most immunogenic, we also discovered that multiplexing beyond four antigens introduces detectable reactogenicity, indicating that there is an upper limit to the number of antigens that can be safely included in a multivalent Q-fever vaccine. *C. burnetii* LPS also demonstrates efficacy as a vaccine antigen in conferring protection in an otherwise monovalent vaccine formulation, suggesting that its addition in multivalent vaccines, as demonstrated by a quadrivalent formulation, would improve protective responses.

## 1 Introduction

*Coxiella burnetii* causes Q fever, which in many cases results in an acute febrile illness but may also manifest chronically and lead to pneumonia and endocarditis, which can be fatal. Owing to its very high infectivity with an ID50 (infectious dose) of 1, high stability in the environment, and aerosol transmissibility, *C. burnetii* is considered a potential biological weapon and classified by the Centers for Disease Control and Prevention as a Tier 2 Select Agent. Q-VAX is a purified suspension of formalin-inactivated *C. burnetii* Phase I Henzerling strain (RSA 331) grown in the yolk sacs of embryonated eggs, which provides robust protection of humans against Q fever (1, 2). However, the vaccine can be reactogenic in individuals who have been previously exposed to *C. burnetii*. Prior exposure must be ascertained by serological screening and intradermal skin testing before immunization, which creates added costs and delays to the vaccination process. Understanding the mechanisms of vaccine-induced protective immunity and minimizing components that can elicit reactogenicities are necessary to rationally design a safe and effective vaccine.

Subunit vaccines, although less immunogenic than whole-cell vaccines (WCVs), are safer and have fewer unknown components. However, they must be delivered with potent adjuvants that enhance antigen recognition, uptake, and processing by antigen-presenting cells (3). Toll-like receptor (TLR) agonists represent the next generation of adjuvants, and several have recently been licensed for clinical use (4–6). The TLRs themselves are a class of pattern recognition receptors (PRRs) commonly found either on the surface or on endosomal membranes of innate immune cells, fibroblasts, and epithelial cells. TLR agonists can engage in downstream signaling to induce the activity of the transcription factor NFκB and the production of cytokines, chemokines, and type I interferons. In this study, we have used a previously described combination adjuvant, IVAX-1, that comprises TLR4 agonist monophosphoryl lipid A (MPLA), TLR9 agonist CpG oligodeoxynucleotide (ODN) 1018, and a squalene emulsion, AddaVAX (7). MPLA is a significantly less toxic synthetic derivative of *Salmonella minnesota* lipopolysaccharide (LPS) due to the removal of one or more acyl chains and phosphate groups (8). MPLA primes adaptive immunity and has been approved for use in humans in the commercial Shingrix and Cervarix vaccines for varicella zoster and Human Papillomavirus (HPV), respectively (8, 9). CpG ODNs mimic bacterial nucleic acids and strongly promote the development of Th1 cells and have been licensed in the Heplisav-B vaccine for hepatitis B (10). AddaVAX is a squalene oil-in-water emulsion that elicits a mixed Th1/Th2 response by antigen presentation, facilitating the transport of antigens, activating immune cells, and inducing cytokine production (11, 12). Similar nano-emulsion adjuvants, including MF59 and AS03, have been used in influenza vaccines (13, 14).

Due to the high-risk factor and labor-intensive process of producing WCVs, *C. burnetii* is an ideal candidate for the development of subunit vaccines. Unlike viruses with small genomes composed of a few proteins, *C. burnetii* Nine Mile phase I (NMI) (RSA 493) has a large proteome of 1,815 annotated proteins to select from, covering a wide variety of antigens in its unconventional life cycle and aiding in host evasion (15, 16). We hypothesize that a multivalent vaccine combining several immunogenic antigens would be the optimal course of action and offer better protection than a monovalent vaccine. Multiple proteins containing multiple epitopes can induce different populations of immune cells for breadth and more closely simulate Q-VAX, which is well-documented to provide lifelong protection (17). These epitopes are recognized by T cells and B cells and are presented on the surface of antigen-presenting cells after internalization and digestion (18). Single-epitope peptides have consistently demonstrated low efficacy, so there has been a push for multivalent constructs. Multiple epitopes allow for binding at multiple sites, which increases downstream effects including simultaneous activation of cellular and humoral responses (19). Antigen selection must fulfill multiple criteria, including expression on the bacterial surface for antibody recognition, availability of processed peptides by major histocompatibility complex (MHC) molecules, and structural properties compatible with efficient protein production (20). In this study, we have taken the whole-antigen approach to activate both B and T cell components of the adaptive immune response (21). We first identified immunogenic *C. burnetii* antigens for use in a multivalent vaccine by using a literature search and compared with published and unpublished protein microarray data. These were then tested by administration of IVAX-1 and other TLR agonists for immunogenicity studies in C57BL/6 mice and validated in an aerosol challenge model using mice and Hartley guinea pigs. We show that several vaccine candidates containing the most immunogenic antigens provide protection against challenge comparable to Q-VAX. Our data from these studies show that our multivalent vaccines induce potent humoral and cellular immune responses in animal models.

## 2 Materials and methods

### 2.1 Reagents and biologics

Q-VAX^®^ was purchased from Seqirus (bioCSL, Melbourne, Australia). Equivalent *C. burnetii* WCV was generously provided by the Dr. James Samuel lab at Texas A&M University. *C. burnetii* NMI clone 7 (RSA 493) was grown in selective ACCM-2 media and inactivated with 2% formalin for 48 h for the WCV. Recombinant proteins were expressed in *Escherichia coli* BL21 cells and purified by multi-column chromatography and endotoxin removal procedure (<0.1 EU/ng) by GenScript (Piscataway, NJ, USA). *C. burnetii* NMI RSA 493 was purchased from BEI Resources (Manassas, VA, USA) and quantified *via* qPCR with genomic DNA extraction *via* High Pure PCR Template Preparation Kit (Roche, Basel, Switzerland) and PowerUp SYBR Green Master Mix (Thermo Fisher Scientific, Waltham, MA, USA) for infection. Primers amplify a 74-base pair fragment in *C. burnetii* com1 [FAF216: 5’ GCACTATTTTTAGCCGGAACCTT 3’, RAF290: 5’ TTGAGGAGAAAAACTGGATTGAGA 3’] (22). AddaVAX™ (squalene oil-in-water emulsion) and CpG ODN 1018 (TLR9 agonist) were purchased from InvivoGen (San Diego, CA, USA) and Integrated DNA Technologies (Coralville, IA, USA), respectively. CpG-ODNs were dissolved in sterile water at 1 mM as stock. MPLA (a TLR4 agonist) was purchased from Avanti Polar Lipids Inc. (Alabaster, AL, USA). TLR7 agonist 2Bxy, an imidazoquinoline derivative, was purified by either high -performance liquid chromatography or gel extraction and confirmed by MALDI-TOF or electrospray ionization-mass spectrometry (23).

### 2.2 Animals

C57BL/6 female mice (6–12 weeks) and Hartley guinea pigs (300–400 g) were obtained from Charles River Laboratories (Wilmington, MA, USA). Animal experiments were approved by the Institutional Animal Care and Use Committee (IACUC) of the University of California, Irvine, and the Animal Care and Use Review Office (ACURO) of the U.S. Army Medical Research and Materiel Command (USAMRMC). Mice were housed in standard cages with enrichment at Animal Biosafety Level 2 (ABSL-2), and guinea pigs were housed in approved ABSL-3 facilities. For immunogenicity studies, mice were anesthetized in induction chambers with inhaled isoflurane/O_2_ and passive scavenging with F/air canisters. Guinea pigs were anesthetized with an intraperitoneal (IP) injection of 100 mg/kg ketamine and 10 mg/kg xylazine in phosphate-buffered saline (PBS). Where indicated, mice were immunized either subcutaneously (SC) at the base of the tail or intramuscularly (IM) in the semitendinosus and semimembranosus muscles of the hind limb. Guinea pigs were immunized intramuscularly in the caudal thigh. Blood was collected from the submandibular vein in mice and the lateral saphenous vein in guinea pigs with 25 G hypodermic needles (Medline, Northfield, IL, USA), and collected into Microvette CB 300 lithium heparin (Sarstedt, Newton, NC, USA), and BD Microtainer PST tubes with lithium heparin (BD, Franklin Lakes, NJ, USA), respectively.

### 2.3 Serological profiling by protein microarrays

Gene identification and nomenclature are based throughout this study on the complete genome sequence published by Seshadri et al. (24). *C. burnetii* proteome microarrays were produced as described previously (25–27). Briefly, proteins from the *C. burnetii* NMI strain RSA 493 proteome were expressed from purified plasmids in an *E. coli*-based cell-free *in vitro* transcription translation system (IVTT) (Biotechrabbit GmbH, Hennigsdorf, Germany). IVTT reactions were printed onto nitrocellulose-coated glass AVID slides (Grace Bio-Labs Inc., Bend, OR, USA) using an Omni Grid 100 microarray printer (Genomic Solutions). Plasma was diluted 1:100 in protein array blocking buffer (GVS, Sanford, ME, USA) and incubated with 0.1 mg/ml of a His-tag-containing peptide (HHHHHHHHHHGGGG) (Biomatik, Wilmington, DE, USA) at room temperature for 30 min to block anti-His antibodies generated by the immunizations. Afterward, the arrays were incubated overnight at 4°C with gentle rocking. Arrays were washed three times with TBS-0.05% Tween 20 (T-TBS) and then incubated with goat anti-mouse immunoglobulin G (IgG)-biotin, IgG1-biotin, or IgG2c-biotin (1:200 in array blocking buffer) (Jackson ImmunoResearch, West Grove, PA, USA) for 1 h at room temperature with gentle rocking. Following another set of T-TBS washes, bound antibodies were detected with streptavidin-conjugated Qdot^®^655 or Qdot^®^800 (Thermo Fisher Scientific, Waltham, MA, USA), diluted 1:250 in array blocking buffer for 1 h at room temperature with gentle rocking. Arrays were washed three times with T-TBS, and the slides were rinsed thoroughly with water and then air-dried by centrifugation at 500 g for 10 min. Images were acquired, and spot fluorescence intensities were quantified using the ArrayCAM™ Imaging System (Grace Bio-Labs, Bend, OR, USA). Signal intensities (SIs) for each antigen on the array were background-corrected by subtracting sample-specific T-PBS buffer signals from purified protein spot signals.

### 2.4 IVTT expression and capture on Sepharose beads

Down-selected proteins were expressed *in vitro* in 200 μl IVTT reactions as described above. Each protein is expressed with an N-terminal 10× polyhistidine and C-terminal HA epitope tags. After 16 h reaction at 21°C, proteins were captured *via* ×10 His tags by adding 100 μl to nickel-charged His-TrapSpin resin (GE Healthcare Life Sciences). The resin consists of ~34 μm-diameter Sepharose beads with a binding capacity of 750 μg of His-tagged protein per ml. Flow-through was reapplied to columns and then washed to remove non-bound IVTT material. The successful capture of proteins to the beads was monitored by printing microarrays of IVTT, flow-through, and bead wash on nitrocellulose-coated slides **(** **Supplementary Figure S2**).

### 2.5 T cell recall assays

Spleens were harvested from mice 10 days after they were boosted *via* the IP route, and erythrocyte-depleted splenocyte suspensions were prepared for T cell recall assay (IFNγ ELISpot) as previously described (28). Purified antigens were titrated in the assay (final concentrations of 10, 5, 2.5, and 0 μg/ml). Spleen cells from naive mice were assayed in parallel as a control for potential mitogenic activity of the recall antigens. Assays were performed in T cell medium (TCM) comprising Iscove’s Modified Dulbecco’s Medium (IMDM), containing 5 × 10^-5^ M β-mercaptoethanol, 100 IU/ml penicillin, 100 μg/ml streptomycin, and 10% heat-inactivated fetal calf serum. After 18 h of incubation, the assay supernatants were collected for multiplex cytokine screening using the LEGENDplex kit (BioLegend Inc., San Diego, CA, USA) according to the manufacturer’s instructions before the ELISpot was processed. Spots were quantified in an ImmunoSPOT^®^ ELISpot plate reader (Cellular Technology Limited, Cleveland, OH, USA).

### 2.6 Guinea pig reactogenicity experiments

Guinea pigs were sensitized with an SC administration of Q-VAX and rested for 2 weeks. RFID microchip Transponders (BMDS Avidity Science, Waterford, WI, USA) were delivered SC for identification and to monitor weight and temperature change. To elicit hypersensitivity responses, four or six approximately 2–3 cm areas were shaved using electric clippers on the right and left flanks. Vaccine candidates, WCV, or PBS sham was then injected intradermally at each of the shaved sites. Temperature, weight, and reaction sites were monitored daily for 2 weeks. Each vaccine candidate was evaluated in four guinea pigs.

### 2.7 Histopathology

Skin sites from guinea pigs were fixed in 10% neutral buffered formalin for at least 72 h at room temperature. For the hypersensitivity experiments, three sections were cut from the shaved areas containing the epidermis to the underlying abdominal or intercostal muscle. Tissues were submitted to AML Laboratories (Jacksonville, FL, USA) for processing, embedding, and sectioning at 5 μm before staining with hematoxylin and eosin (H&E). Histopathology slides were deidentified and evaluated by an American College of Veterinary Pathologists (ACVP) board-certified pathologist. Histopathologic scoring was performed on a 0–5 scale.

### 2.8 Guinea pig challenge experiments

Guinea pigs were SC administered RFID microchip transponders in the back of the neck for identification purposes and to monitor weight and temperature for the duration of the experiment. They were then administered candidate vaccines, WCV, or PBS (sham) in 100 -μl sterile PBS by intramuscular injection in the semitendinosus and semimembranosus muscles. A boost vaccine was given in the opposite hind limb 2 weeks later. Blood and plasma were collected from the lateral saphenous vein on days 10, 21, 28, 35, and 42 post-prime. Guinea pigs were intratracheally infected with 5 × 10^5^ genomic equivalents (GEs) of *C. burnetii* NMI RSA 493 after resting for 7 weeks post-prime. For this, animals were anesthetized with an IP injection of 100 mg/kg ketamine and 10 mg/kg xylazine in PBS. A MADgic pediatric laryngotracheal mucosal atomizer device (Teleflex, Morrisville, NC, USA) was inserted to administer the bacteria intratracheally in 100 μl of PBS. Guinea pigs were monitored daily, and weight and temperature measurements were taken. Four to five guinea pigs were utilized for each experimental group.

### 2.9 Statistical analyses

Statistical analyses were performed with GraphPad Prism v9.4.0 (GraphPad Software, La Jolla, CA, USA). Results were compared using one-way or two-way ANOVA with Dunnett’s or Tukey’s correction for multiple comparisons. Differences were considered significant if p-value ≤ 0.05 (*), ≤ 0.01 (**), ≤ 0.001 (***), or ≤ 0.0001 (****).

## 3 Results

### 3.1 Identification of target antigens in the response to Q-VAX^®^

**Figure 1:**
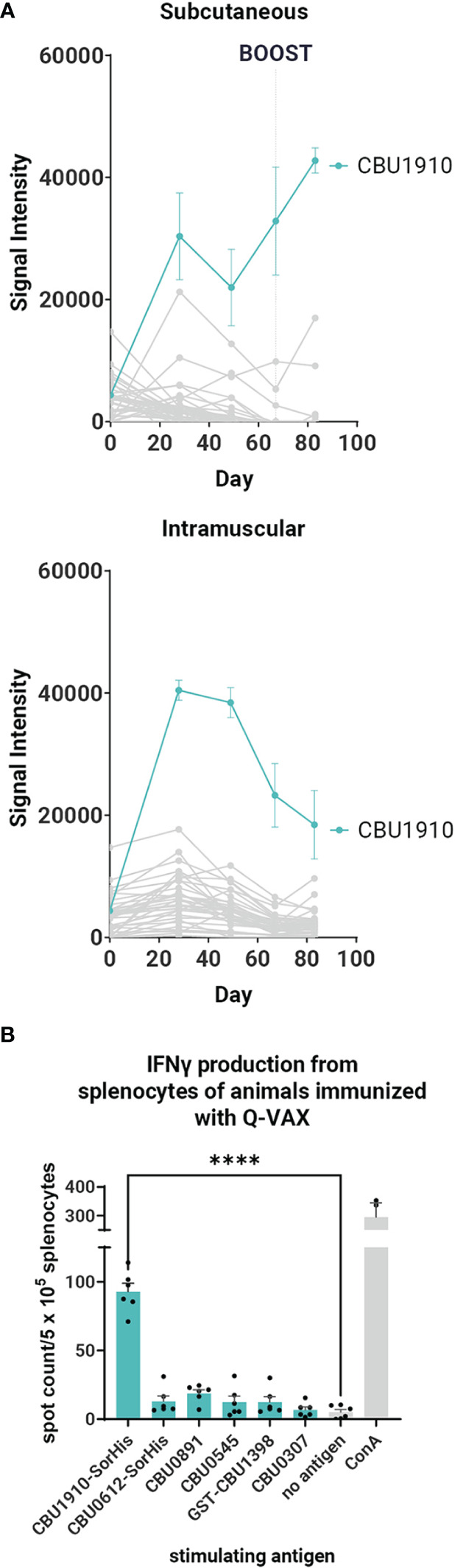
Antibody and T cell profiling after administration of Q-VAX^®^. **(A)** Time course IgG profiles from plasma of C57BL/6 mice administered Q-VAX *via* subcutaneous and intramuscular routes (n = 5 mice per group). The top 5 Tier 1 antigens and the top 30 reactive antigens on d28 are included. The hashed line in panel A indicates when the boost was administered (d69); mice in the intramuscular group were not boosted. **(B)** In T cell recall experiments, C57BL/6 mice (n = 5) were immunized with Q-VAX^®^ intraperitoneally, and their splenocytes were harvested 10 days after the prime. The splenocytes were stimulated by purified protein from the downselected panel of lead antigens. Significance is compared to the no -antigen group. Statistics were performed with one-way ANOVA and Dunnett’s multiple comparison test. p-value ≤ 0.0001 (****).

It was of considerable interest to first profile antibodies (Abs) and T cells in response to Q-VAX to identify recognized antigens as subunit vaccine candidates. Ab (IgG) profiling was performed in C57BL/6 mice administered a single dose *via* SC or intramuscular routes using proteome microarrays (**Figure 1A**). Plasma was collected on days 0, 28, 49, and 69. Only one protein, CBU1910, was consistently recognized, with other reactive proteins giving a scattered, more stochastic recognition pattern. The breadth of the Q-VAX Ab profile was unexpectedly narrow, given that there are 1,815 different proteins in the proteome. There were no obvious differences in Ab profiles between the intramuscular and SC routes of immunization.

To attempt to broaden the Ab profile and identify additional candidate vaccine targets, mice primed *via* the SC route were boosted with Q-VAX, weighed, and carefully observed daily for changes in behavior. No adverse events were seen, and animals were bled at the end point 14 days later (day 83). After boosting, IgG signals increased only to existing reactive antigens, namely, CBU1910, with no increase in breadth of response (**Figure 1A**). These data show that the antibody breadth in response to administration of Q-VAX is remarkably narrow, even after boosting.

Given the limited serological breadth induced by Q-VAX, we broadened the search to include serological profiling in cases of natural infection. For this, we used published and unpublished in-house protein microarray studies from human and animal Q-fever studies as well as mass spectrometry identification of seroreactive proteins from *C. burnetii*, as reviewed in 2013 by Vranakis et al. (25, 26, 29–34). Antigens were ranked based on reactivity on the protein microarrays, and a total score was assigned based on these two metrics (**Table 1**). Higher priorities were given to those antigens with a predicted transmembrane domain, as these are more likely to be surface proteins and have easily accessible epitopes for antigen presentation. This list initially comprised CBU0612, CBU1910, CBU0891, CBU0307, and CBU0664 as “Tier 1” antigens.

**Table 1:** Literature, proteomics, and protein microarray selection of *Coxiella burnetii* protein antigen candidates.

Tier	ORF	Gene ID	Description	MWt (kDa)	Array studies (n=13)	2D gel/MS (n=10)	Overall score	Immuno 2D gel/MS	TM HMM	Literature	Immunogenicity	Final subset	Tags and mods
1	CBU1910	com1	Outer membrane protein	27.7	8	9	17	yes	yes	yes	yes	yes	SorHis, Truncated
1	CBU0612	skp	Outer membrane chaperone OmpH	18.2	9	9	18	yes	yes	yes	yes	yes	SorHis, Truncated
1	CBU0891		hypothetical exported membrane associated protein	34.3	8	4	12	no	(yes)	yes	yes	yes	Truncated
1	CBU0307		OmpA-like transmembrane domain-containing protein	25.4	2	6	8	yes	no	yes	yes	yes	Truncated
1	CBU0664		ISAs1 family transposase	41.9	1	0	1	no	yes	no	no	no	
2	CBU0092	ybgF	tol-pal system protein YbgF	33.5	6	9	15	yes	no	yes	no	no	
2	CBU0545	lemA	LemA family	23.6	5	8	13	no	yes	yes	yes	yes	Full length
2	CBU1398	sucB	2-oxoglutarate dehydrogenase E2	44.5	6	6	12	yes	no	yes	yes	yes	GST
2	CBU0630	mip	peptidyl-prolyl cis-trans isomerase	25.3	3	8	11	yes	no	yes	yes	no	GST
2	CBU1143	yajC	preprotein translocase subunit	12.8	5	6	11	yes	(yes)	yes	no	no	
2	CBU0718		Hypothetical membrane associated protein	10.3	3	7	10	no	no	no	yes	no	GST
2	CBU1627	IcmE	Type IV (Icm/Dot) secretion system protein	114.3	5	4	9	no	yes	yes	no	no	
2	CBU1094		Membrane fusion protein, multidrug efflux system	41.8	3	4	7	no	yes	no	yes	no	His-GST
2	CBU1513		short chain dehydrogenase/reductase oxidoreductase	28.4	5	1	6	no	no	no	yes	no	GST
2	CBU0611		Hypothetical Outer Membrane Protein insertion porin family	88.3	0	4	4	no	yes	yes	yes	no	His-GST
2	CBU1260	ompA	OmpA-like transmembrane domain-containing protein	27.3	0	4	4	yes	yes	yes	no	no	
2	CBU0198		Hypothetical Outer Membrane Protein insertion porin family	63.6	0	3	3	no	yes	yes	no	no	

Properties of the identified proteins from publicly available datasets and databases are noted. Includes columns indicating use and modifications in experiments. “Immunogenicity” indicates the protein’s use in early T cell screens.

Where possible, we attempted to produce “tag-less” proteins to minimize incorporation of off-target epitopes. Thus, CBU0891, CBU0307, and CBU0664 proteins were expressed with the SUMO-His tag that was cleaved from the protein before use (35). However, CBU0612 and CBU1910 were expressed with Sortase recognition motifs (either C-terminal LPXTG Sortag or an N-terminal GGG tag) with the intention of using the Sortase tag to couple proteins to TLR agonists (36). The expression and solubility of CBU0891 and CBU0307 were initially low but improved by subsequently expressing truncated versions lacking transmembrane domain and signal peptide, and signal peptide, respectively. CBU0612-Sortag and CBU1910-Sortag were subsequently modified with a C-terminal polyhistidine (termed CBU0612-SorHis and CBU1910-SorHis) to evaluate the utility of tris-NTA for coupling proteins to TLRs in place of Sortase (37). Overall, proteins expressed with His-SUMO (CBU0891, CBU0307) showed only modest solubility. Protein CBU0664 showed poor solubility and was not pursued further.

An additional 12 antigens (Tier 2) were selected and tested in pilot experiments for *E. coli* expression and solubility. All 12 Tier 2 proteins were expressed as GST-GG fusion proteins separated by a TEV protease cleavage site to improve solubility. The GG motif was included, so cleavage of His-GST-GG-TEV yields protein with an N-terminal GGG tag available for downstream coupling reactions. Pilot studies showed that only HisGST-CBU0545 remained soluble after GST cleavage. Of the 12 Tier 2 proteins, seven were suitable for scale -up (CBU0545, GST-CBU1398, GST-CBU0630, GST-CBU0718, HisGST-CBU1094, GST-CBU1513, and HisGST-CBU0611), whereas five proteins remained either poorly expressed or insoluble (CBU0092, CBU1143, CBU1627, CBU1260, and CBU0198) and were not investigated further. The final list of purified Tier 1 and Tier 2 proteins used for immunogenicity studies is indicated in **Table 1**.

To determine if Q-VAX induced a T cell response against any of the purified Tier 1 and Tier 2 proteins, we performed a T cell recall assay on splenocytes of mice that were immunized IP with Q-VAX. Splenocytes were harvested 10 days after immunization, pooled from n = 3 mice, and incubated with purified recall protein for 18 h (**Supplementary Figure S1**). Antigen-specific IFNγ responses were seen to GST-CBU1398, CBU0891, CBU0545, and CBU1910-SorHis, with CBU1910-SorHis being dominant. Antigens that induced low levels of IFNγ in the T cell recall response and were difficult to purify were excluded from further experiments. The T cell recall experiment was repeated with a subset of six lead candidate antigens (**Table 1**), with a representative experiment shown in **Figure 1B**. Overall, the data indicate that CBU1910 is dominant in the antibody response to Q-VAX, which is accompanied by a robust IFNγ response in the recall assay (p < 0.0001).

### 3.2 Individual antigens are immunogenic when administered to mice

**Figure 2:**
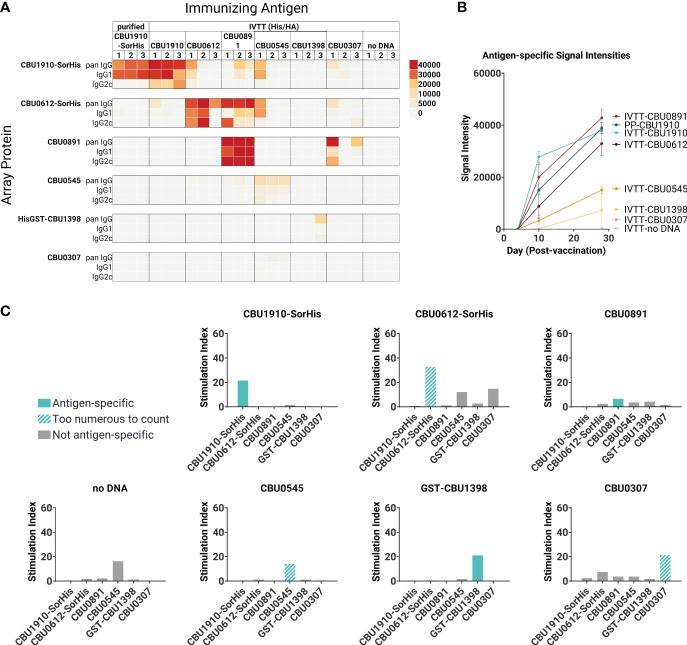
Immunogenicity screen of candidate (*C*) *burnetii* antigens. Groups of C57BL/6 mice (n = 3 per group) were immunized with purified CBU1910-SorHis protein (pp1910) or His-Trap resin to which different (*C*) *burnetii* proteins expressed in IVTT reactions were bound *via* polyhistidine tags and formulated in AddaVAX™ for immunization. **(A)** Plasma was probed on microarrays displaying purified (*C*) *burnetii* proteins, and bound Abs were visualized with secondary Abs against IgG, IgG1, and IgG2c. Heat map shows signals on d28; red = high, yellow = intermediate, white = low; arrayed proteins were listed on the left and immunizing antigens were listed at the top. **(B)** Array signals (group mean ± SD) at different time points post-immunization. **(C)** T -cell immunogenicity screen of candidate (*C*) *burnetii* antigens. Groups of mice (n = 3) were immunized with IVTT-expressed proteins and boosted 8 weeks later with purified proteins indicated in each panel for the recall assay (IFNγ ELISpot). Numbers of spot-forming cells at different concentrations of antigen are expressed as a fold-over the number of spots at 0 mg/ml antigen. Colored bars = assay recall antigen corresponding to immunizing antigen.

In parallel with protein purification described above, immunogenicity studies were also performed with antigens expressed from pXi plasmids in transcription/translation (IVTT) reactions (as used for custom protein microarrays) and captured onto Ni-Sepharose beads. This method, which exploits the ability of IVTT to express any protein in soluble form and obviates the need for traditional protein purification, allows many potential target antigens to be screened directly for immunogenicity *in vivo*. Loading of protein to the beads was confirmed by printing the protein beads on arrays and probing arrays with anti-epitope tag antibodies (**Supplementary Figure S2**). We then administered each individually formulated with AddaVAX to mice (n = 3 per group) *via* the SC route. One group of animals was administered purified CBU1910 protein for comparison. Plasma was collected at days 0, 10, and 28 and analyzed for antigen-specific IgG responses on protein microarrays displaying both IVTT-expressed and purified proteins, as shown in **Figures 2A, B**. Robust antibody responses were evident to three of the proteins tested —CBU1910, CBU0612, and CBU0891. The Abs produced against the IVTT expression products were both IgG1 and IgG2c, while those engendered by purified protein (CBU1910-SorHis) were IgG1 (Th2) only. Increased IgG2c responses correspond to the Th1 T cell response, which is defined by proinflammatory cytokine production and macrophage activation promoting intracellular bacterial killing (38). IgG1 corresponds to a Th2 response, which is appropriate for upregulating granule release in basophils and other granulocytes. Previous studies have demonstrated that protective immunity against *C. burnetii* is driven by a Th1 response, as evident by elevated IFNγ, TNFα, and IgG2c antibodies (39–41). We attribute this to the purified proteins lacking immunostimulatory components when compared to the IVTT expression products. The increase in IgG signals over time is shown in **Figure 2B**.

A T cell recall assay was performed with the same mice boosted with the corresponding adjuvanted purified antigens *via* the IP route on day 28, and spleens were harvested 10 days later (**Figure 2C**). Minor nonspecific or mitogenic background activity was noticed against purified CBU0545 in splenocytes from mice immunized with beads incubated in IVTT without DNA template; the remaining proteins elicited no response. Antigen-specific IFNγ responses were seen to all the priming antigens except for CBU0891. In the case of CBU0545, the recall response remained high at all antigen concentrations tested, revealing a robust antigen-specific response above any nonspecific effects. The weakest IFNγ response was seen against CBU0891. T cell cross-reactivities were noted; for example, mice immunized with CBU0612-SorHis showed measurable cross-reactivity for CBU0545 and CBU0307. These were non-reciprocal and appeared independent of shared tags, and sequence alignments did not reveal any obvious reasons for these patterns. Nevertheless, the serology and T cell data were consistent with the immunogenicity of all eight proteins when administered individually. Overall, the data indicate that the candidate vaccine antigens can engender Ab and/or T cell responses in C57BL/6 mice.

### 3.3 IVAX-1 is a potent adjuvant and increases Th1 and IgG2c responses

**Figure 3:**
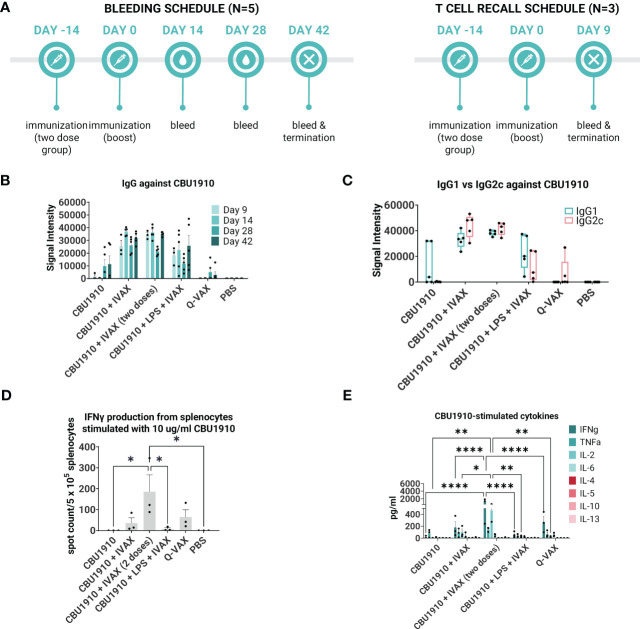
IVAX-1 (MPLA, CpG1018, AddaVAX) is a potent combination adjuvant for enhancing IgG2c responses and increasing proinflammatory cytokine production. **(A)** Timeline of events. Two groups with the same immunizations composed of immunodominant protein CBU1910 and adjuvant combinations were run simultaneously. One group (n = 5) was used to study the longevity of the IgG response, and another (n = 3) was used for T -cell assays. **(B)** Plasma from both experimental groups was probed on a *C. burnetii* protein microarray looking at the response to immunizing antigen CBU1910. Q-VAX and PBS were used as positive and negative controls. **(C)** Plasma from day 42 was used to assess IgG1 and IgG2c responses on the protein microarray. **(D)** Animals from the T -cell recall group were terminated on day 9, and their splenocytes were subjected to stimulation for 18 h with CBU1910. Anti-IFNγ capture antibodies were used to determine spot counts in an IFNγ ELISpot. Statistics were performed with one-way ANOVA and Dunnett’s multiple comparison test. **(E)** Supernatants from the 18-h stimulation were assessed for Th1/Th2 cytokines using a cytokine bead assay. Extrapolated values for the PBS control group were subtracted from the other groups. Statistics were performed with two-way ANOVA and Tukey’s multiple comparison test. p-value ≤ 0.05 (*), ≤ 0.01 (**), or ≤ 0.0001 (****).

We next evaluated the immunogenicity-enhancing effects of including TLR agonists and the squalene oil-in-water emulsion AddaVAX. For this, we formulated purified CBU1910 in a combination adjuvant, IVAX-1, which comprises of TLR4 agonist MPLA, TLR9 agonist CpG oligodeoxynucleotide (ODN) 1018, and AddaVAX (7, 42). Formulations containing purified CBU1910 and IVAX-1 were administered to C57BL/6 mice as a single dose and compared to control groups Q-VAX and PBS (**Table 2** and **Figure 3A**). One group was given two doses 14 days apart to observe the benefits of a prime/boost model versus a single dose. Interestingly, including a boost did not significantly increase IgG responses when compared to single-dose groups. Another experimental group contained NMI LPS to emulate Q-VAX more closely. LPS has been shown by other groups to be a prospective antigen, with adoptive transfer of immune sera or splenocytes protecting naive mice (43).

**Table 2:** Vaccine formulations including adjuvants for immunogenicity studies in C57BL/6 mice.

Group	Name	Antigen	Adjuvants
**1**	CBU1910	Purified SorHis-tagged truncated CBU1910 protein	–
**2**	CBU1910 + IVAX-1	Purified SorHis-tagged truncated CBU1910 protein	MPLA, CpG1018, AddaVAX
**3**	CBU1910 + IVAX-1 (2 doses)	Purified SorHis-tagged truncated CBU1910 protein	MPLA, CpG1018, AddaVAX
**4**	CBU1910 + LPS + IVAX-1	Purified SorHis-tagged truncated CBU1910 protein	NMI LPS, MPLA, CpG1018, AddaVAX
**5**	Q-VAX	Formalin-inactivated whole -cell vaccine positive control	–
**6**	PBS	PBS negative control	–

Candidate vaccines contained 3 μg of antigen per vaccine dose. For adjuvants, AddaVax was dosed at 50% v/v, 3 nmol MPLA, and 1 nmol CpG-1018. Q-VAX was dosed at 2.5 µg of antigen per vaccine dose.

Plasma was collected on days 9, 14, 28, and 42 and evaluated on protein microarrays for antibodies against CBU1910 (**Figure 3B**). Interestingly, IgG signals had lower intensities in the group containing the NMI LPS. Plasma from day 42 was analyzed for IgG1 and IgG2c to determine Th2 vs. Th1 responses, respectively (**Figure 3C**). Mice immunized with CBU1910 without adjuvant skewed very heavily toward an IgG1/Th2 response, while two of the five animals in the Q-VAX group polarized toward IgG2c/Th1. Experimental groups composed of CBU1910 with adjuvants IVAX-1, IVAX-1 prime/boost, or IVAX-1 + NMI LPS exhibited a balanced IgG1/IgG2c response and higher overall responses when compared to the unadjuvanted group (p = 0.0151, p = 0.0008, and p = 0.7565 for IgG1; p < 0.0001, p < 0.0001, and p = 0.4710 for IgG2). We observed a significant dampening response with lower IgG2c signal intensities in the group containing the NMI LPS when compared to the CBU1910 + IVAX-1 group (p < 0.0001).

In the T cell immunogenicity screen, animals were primed with a single dose and euthanized 9 days later. One group was administered a boost 14 post-prime for comparison (**Figure 3A**). Splenocytes were stimulated for 18 h with CBU1910 antigen, and IFNγ produced from the stimulation was captured and quantified by both ELISpot (**Figure 3D**) and cytokine bead assay (**Figure 3E**). The prime/boost group with CBU1910 and IVAX-1 generated more IFNγ spot-forming cells than the other groups (p = 0.0380), and the group given only the protein antigen by itself generated 0 spots. The addition of IVAX-1 to antigens trended toward an increase in IFNγ production when compared to antigen alone (p = 0.9806), although the addition of NMI LPS seemed to dampen the response, similar to the IgG response. The response of the adjuvanted group with NMI LPS is weaker than that of Q-VAX (p = 0.8776). The cytokine bead assay results also reflected the IFNγ T -cell recall results in that the group that received a prime/boost of CBU1910 and IVAX-1 adjuvant induced the highest IFNγ and IL-2 response, corresponding to a Th1 response (**Figure 3E**). IFNγ and IL-2 production was significantly higher in the prime/boost group compared with the single-dose immunization (p < 0.0001 and p = 0.0186, respectively). The Th2 cytokine levels generated in response to recall antigen stimulation in all cases were low. Overall, these data show that the addition of the IVAX-1 adjuvant enhanced the IgG, T cell recall, and cytokine response when compared to soluble antigen alone and sham-vaccinated groups. The inclusion of NMI LPS lowers immunogenicity in reducing IgG and IFNγ production.

### 3.4 Guinea pig reactogenicity model establishes reactogenicity threshold with multivalency

The Hartley guinea pig is considered a more relevant animal model than the mouse for the development of Q-fever vaccines due to its high susceptibility to respiratory pathogens with its ability to develop fever and other visible pathological changes (44–46). The formulations used in the guinea pig immunogenicity studies are indicated in **Table 3**. Group 1 contains TLR7 agonist 2Bxy and was a formulation from a previous study (27). 2Bxy is an imidazoquinoline derivative that promotes CD8^+^ T cell activity (47). We evaluated reactogenic responses to the vaccine candidates using an intradermal assay in guinea pigs. For this, guinea pigs were sensitized to Q-VAX *via* SC injection followed by resting for 2 weeks (48, 49). Animals were shaved in their flanks, and vaccine formulations were then administered to the exposed skin. Experimental animals (n = 4) received intradermal immunizations of the six candidate vaccines, and control animals (n = 7) received WCV and PBS immunizations. Weights and temperatures were monitored for 14 days (**Supplementary Figure S3A**). There was no significant difference between experimental and control groups, and all animals steadily gained weight over time.

**Table 3:** Vaccine formulations used in Hartley guinea pig challenge and reactogenicity studies.

Group	Name	Antigen	Adjuvants
1	6 Ag + TLR7 + IVAX-1	CBU1910, CBU0612, CBU0891, CBU0545, CBU0307, CBU1398	2Bxy, MPLA, CpG1018, AddaVAX
2	6 Ag + IVAX-1	CBU1910, CBU0612, CBU0891, CBU0545, CBU0307, CBU1398	MPLA, CpG1018, AddaVAX
3	4 Ag + IVAX-1	CBU1910, CBU0612, CBU0891, CBU0545	MPLA, CpG1018, AddaVAX
4	4 Ag + IVAX-1 + NMI LPS	CBU1910, CBU0612, CBU0891, CBU0545	MPLA, CpG1018, AddaVAX, NMI LPS
5	CBU1910 + IVAX-1	CBU1910	MPLA, CpG1018, AddaVAX
6	CBU1910 + IVAX-1 + NMI LPS	CBU1910	MPLA, CpG1018, AddaVAX, NMI LPS
7	Q-VAX/WCV	Formalin-inactivated whole -cell vaccine positive control	–
8	PBS	PBS negative control	–

Candidate vaccines contained 0.25 nmol per antigen in each vaccine dose. AddaVax was dosed at 50% v/v, MPLA at 2 nmol, CpG-1018 at 2 nmol. Q-VAX was dosed at 5 µg of antigen per vaccine dose.

**Figure 4:**
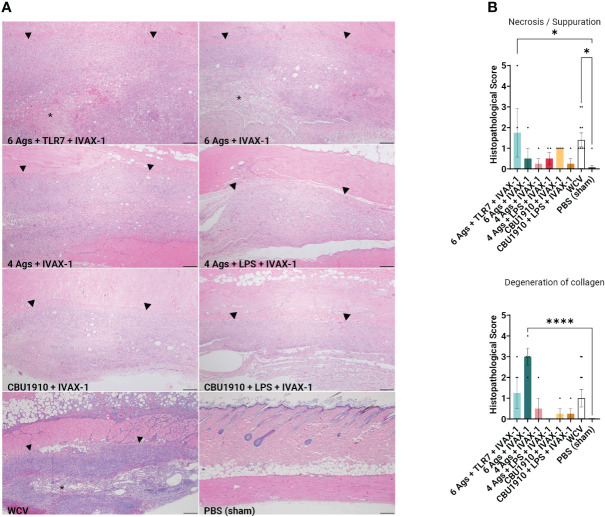
Guinea pig vaccine formulations were evaluated for reactogenicity. All animals were sensitized with Q-VAX and rested for 14 days, then intradermally administered either the six vaccine candidates (n = 4) or Q-VAX and PBS intradermally (n = 7) on shaved skin sections. **(A)** Representative histopathological hematoxylin and eosin (H&E)-stained skin sections of experimental groups at 4× magnification with a 200 -µm scale bar. Arrowheads border areas of immune cell infiltrate/inflammation, and asterisks indicate areas of degenerate neutrophils/abscess formation. **(B)** Mean histopathological scores for experimental groups separated into different morphologic categories. Significance is compared to the PBS group. Statistics were performed with one-way ANOVA and Dunnett’s multiple comparison test. p-value ≤ 0.05 (*) or ≤ 0.0001 (****).

At the end of the 14-day period, the animals were euthanized and the injection sites were sectioned for H&E staining (**Figure 4A**). Pathology in each section was scored on a scale from 0 to 5, with 5 being the most severe. Histopathology evaluation was parsed into four morphological categories: necrosis/suppuration, degeneration of collagen, fibrosis/granulation tissue, and mononuclear cell infiltration (**Figure 4B** and **Supplementary Figure S3B**). Necrosis and suppuration are weighted the most heavily in the parsed scores, with Group 1 and WCV demonstrating the most (p = 0.0253 and p = 0.0149). Group 1, containing six antigens, TLR7 agonist, and IVAX-1, showed marked cellular inflammation composed primarily of macrophages with occasional central foci of necrosis and cellular debris (abscesses) or collagen degeneration. There is a moderate amount of fibrosis surrounding the inflammation, and foci of hemorrhage are present within the lesions. Multifocally, adjacent skeletal muscle myofibers are degenerate. These observations are comparable with findings in the WCV sites. The presence and severity of foci of necrosis in the remaining experimental groups were not significantly greater than those in the PBS group. Group 2 exhibited noticeable degeneration of collagen (p < 0.0001) compared to the PBS control group, while all the remaining experimental groups were comparable to the WCV group. In all experimental groups, there was moderate fibrosis within the subcutis as well as mononuclear cell infiltration (**Supplementary Figure S3B**).

### 3.5 IgG response in plasma is antigen-specific and durable in multivalently immunized Hartley guinea pigs

**Figure 5:**
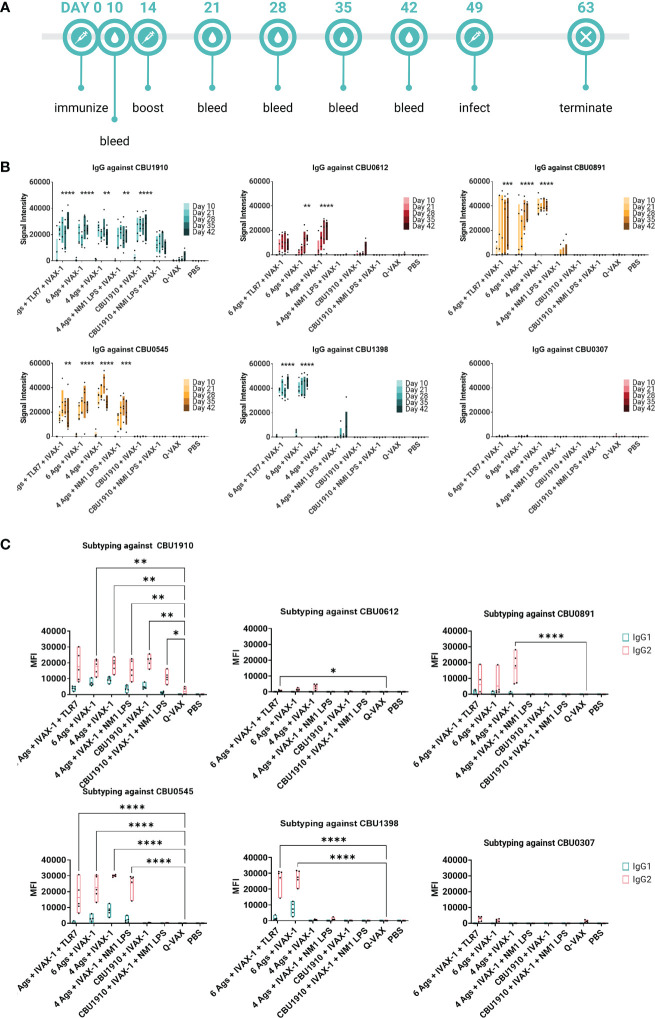
Multiple antigens induce IgG responses in Hartley guinea pigs. **(A)** Timeline of events. Animals were rested for 7 weeks after initial immunization prior to challenge. A challenge study with *Coxiella burnetii* strain NMI RSA 493 was performed in Hartley guinea pigs (n = 5). Formulations with four antigens include CBU1910, CBU0891, CBU0612, and CBU0545. CBU1398 and CBU0307 were included in formulations involving six antigens. NMI LPS used as an immunogen in candidate formulations was extracted from formalin-inactivated *(C) burnetii* NMI RSA 493. TLR7 is 2Bxy and part of the top formulation from a previous challenge study serving as a baseline. IVAX-1 includes MPLA, CpG1018, and AddaVAX. **(B)** Plasma was collected at intervals on days 10, 21, 28, 35, and 42 post-prime and assessed for IgG production using the protein microarray platform containing (*C*) *burnetii* antigens. Significance looks at plasma from day 42 compared to WCV and performed with one-way ANOVA and Dunnett’s multiple comparison test. **(C)** Plasma from day 42 was assessed for the production of IgG1 and IgG2 on the *(C) burnetii* protein microarray. Statistics were performed with two-way ANOVA and Dunnett’s multiple comparison test. p-value ≤ 0.05 (*), ≤ 0.01 (**), ≤ 0.001 (***), or ≤ 0.0001 (****).

We also wanted to confirm whether the antigens identified as immunogenic in the mouse model were similarly immunogenic in the guinea pig. Animals were administered multivalent protein vaccines with the IVAX-1 adjuvant, boosted 14 days later, and rested for 7 weeks. Plasma was collected from vaccinated guinea pigs at regular intervals and assessed on the protein microarray platform for antigen-specific antibody responses (**Figure 5A**). Consistent with mouse studies, Q-VAX generated antibodies to CBU1910 only, which increased over time. All six experimental groups elicited durable CBU1910-specific IgG responses that persisted to day 42 post-immunization (p < 0.0001, p < 0.0001, p = 0.0027, p = 0.0012, p < 0.0001, and p = 0.3828). Interestingly, the introduction of NMI LPS dampened the IgG response to antigens CBU0891, CBU0612, and CBU0545. IgG responses against CBU0307 were not detectable in any of the groups despite being an antigen in two of the six experimental groups. This corroborated with **Figure 2** when mice were immunized with individual antigens. The vaccine formulation group containing only CBU1910 and IVAX-1 exhibited mild cross-reactivity with CBU0612, but this is likely due to the purified proteins containing the same Sortag tag used in the purification process.

IgG1 and IgG2 responses were also measured on a protein microarray to assess Th1/Th2 biases (**Figure 5B**). Based on the subtyping data, all experimental vaccine formulations generated stronger CBU1910-specific IgG2 responses than Q-VAX (p = 0.0692, p = 0.0043, p = 0.0057, p = 0.0070, p = 0.0068, and p = 0.0445). Generally, signal intensities for IgG2 were higher than IgG1, indicating that the formulations all skewed more toward Th1 than Th2 response. Again, introducing the NMI LPS dampened the response for both IgG1 and IgG2 against CBU0612 and CBU0891. Additionally, IgG against NMI LPS was evaluated (**Supplementary Figure S4**). Even though LPS has been added in groups 4 and 6, the only observable response to NMI LPS is from the Q-VAX group, with signals concentrated in IgG2.

### 3.6 Multivalent vaccines demonstrate protection against intratracheal *Coxiella. burnetii* NMI challenge

Guinea pigs used for immunogenicity screening were challenged 7 weeks post-prime with 5 × 10^5^ genomic equivalents of *C. burnetii* NMI RSA 493. Animals were monitored for weight and temperature change over a 14-day period post-infection. WCV (an in-house produced vaccine equivalent to Q-VAX) and sham-PBS were used as positive and negative controls, respectively.

**Figure 6:**
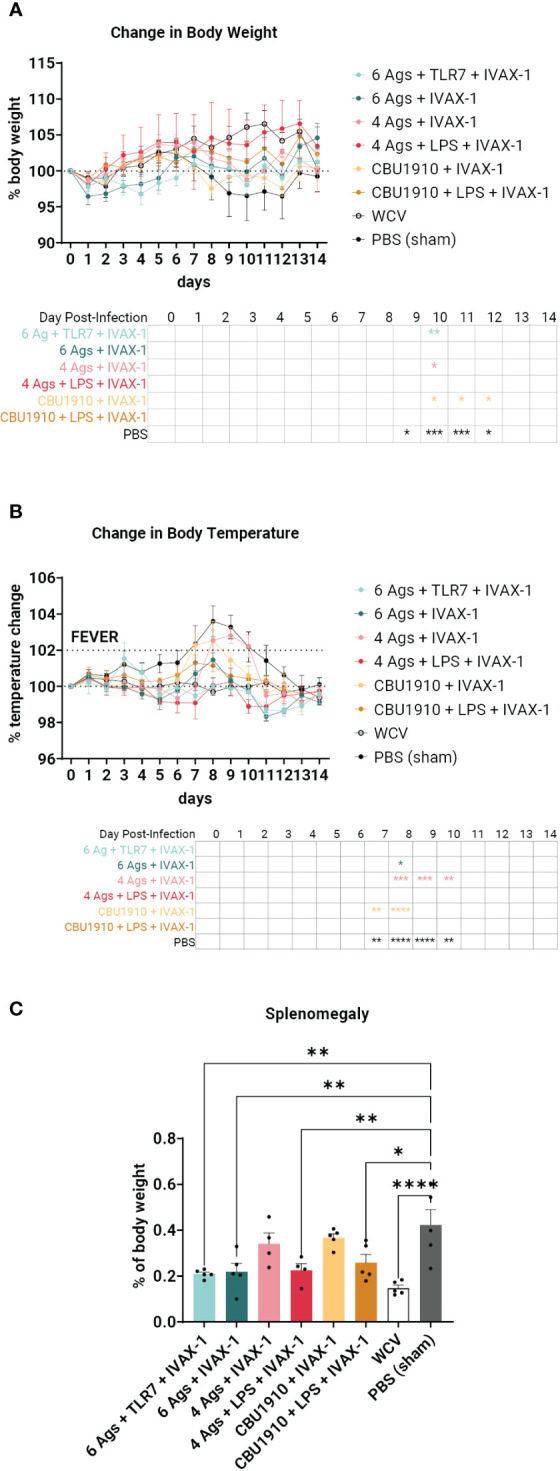
An intratracheal aerosol challenge study with *Coxiella burnetii* strain NMI RSA 493 was performed in Hartley guinea pigs (n = 5). **(A)** Changes in body weight in calculated percentages were recorded for 14 days after infection. Significance is compared to the WCV group and performed with two-way ANOVA and Dunnett’s multiple comparison test. **(B)** Changes in temperature in calculated percentages were recorded for 14 days after infection. Fever is denoted as an increase in temperature greater than 2%. Significance is compared to the WCV group and performed with two-way ANOVA and Dunnett’s multiple comparison test. **(C)** Splenomegaly was determined after termination by comparing to the PBS group. Statistics were performed with one-way ANOVA and Dunnett’s multiple comparison test. p-value ≤ 0.05 (*), ≤ 0.01 (**), ≤ 0.001 (***), or ≤ 0.0001 (****).

Sham-vaccinated animals exhibited a marked decrease in body weight between days 9 and 12 and increased body temperature between days 7 and 10 when compared to the other groups (**Figures 6A, B****)**. WCV positive control animals showed steady increases in body weight and maintained consistent body temperature for the duration of the 2-week challenge. When observing changes in body weight, the groups that performed most similarly to WCV include groups 2, 4, and 6 with no significant difference on any of the days (**Figure 6A**). This includes both groups that include NMI LPS as an antigen. Change in temperature shows groups 1, 4, and 6 with no marked significance on any of the days when compared to WCV (**Figure 6B**). The monovalent group containing NMI LPS, group 6, outperforms the quadrivalent group lacking LPS, group 3, in both changes in body weight and temperature, which exhibits significance on days 10 and 8–10 in those categories, respectively (body weight d10 p = 0.0298; body temperature d8 p = 0.0002, d9 p = 0.0002, and d10 p = 0.0079). Meanwhile, the monovalent group 6 demonstrates no significant difference when compared to WCV for the entire 14-day duration. From this, we can conclude that the groups containing six antigens, groups 1 and 2, showed similar trends with little difference when compared to positive control WCV changes in body temperature and weight, and the inclusion of NMI LPS, in groups 4 and 6, provides significant additional protection.

The animals were terminated after the 14-day observation period. Splenomegaly, a hallmark of Q fever, was significantly lower in four of the six vaccine candidate groups when compared to PBS sham (**Figure 6C**) (p = 0.0013, p = 0.0022, p = 0.5071, p = 0.0055, p = 0.7927, and p = 0.0165). The top performers were groups 1, 2, and 4 and were determined by being the most comparable to WCV. Group 6 also showed a significant reduction in splenomegaly despite containing a single protein antigen.

## 4 Discussion

In this study, we tested the hypothesis that the immunogenicity and efficacy of *C. burnetii* subunit vaccine formulations would increase with antigen multimerization. To address this, we first identified several lead candidate antigens using a proteomic screening approach for antibody and T cell target antigens (**Figure 1**). After undergoing an extensive selection and purification process, we were able to show that a panel of 6 *C. burnetii* antigens was immunogenic individually, capable of eliciting antigen-specific antibody and/or T cell recall responses (**Figure 2**). Use of IVAX-1 as an adjuvant enhanced the Th1 response to match that of protective WCVs such as Q-VAX (**Figure 3**) while also increasing the default Th2 response for an overall balanced profile. We demonstrated efficacy in a Hartley guinea pig model and showed that a multivalent formulation provided the best protective response with some additional protection afforded by including NMI LPS (**Figures 4**–**6**)

The importance of antibody-mediated immunity (AMI) in *C. burnetii* infection has long been a topic of contention. Previous studies have shown that immunized patients seropositive for *C. burnetii* develop IgA- and IgG -specific antibody responses to phase I antigen (50, 51). Acute Q fever patients generate IgM to phase I antigen, while chronic Q fever patients develop IgA and IgG to phase I antigen (52). Other *in vitro* studies demonstrated that incubating *C. burnetii* with immune sera increased its phagocytic uptake by macrophages (53). The consensus is that antibodies affect bacterial uptake by phagocytes in the early stages of infection but have no effect on the replication and growth of internalized *C. burnetii*. A study conducted by Zhang et al. (43) showed that splenomegaly and bacterial burdens in SCID (T and B cell -deficient) mice were not reduced with adoptive transfer of immune sera and B cells. In another study, Read et al. (54) show that SCID mice that were reconstituted with T cells were able to control infection just as well as those that were rescued with both B and T cells. These studies show that even though antibodies, especially those against phase I antigens, are useful diagnostic markers, they appear to play a smaller role in bacterial control and protection against infection. Despite Q-VAX’s ability to provide lifelong protection with a single immunization, its antibody breadth is limited with CBU1910 being the only immunodominant antigen on a whole-proteome microarray (**Figure 1A**). Our immunizing antigens generated improved IgG responses when compared to Q-VAX (**Figures 2**, **3B**, **5B**) and, while not as critical to bacterial elimination, are still valid indicators of immunogenicity and the Th1/Th2 response.

Cell-mediated immunity (CMI) is essential for protection against *C. burnetii*. Studies by other groups have shown that IFNγ and TNFα are critical markers associated with reduced *C. burnetii* burden post-infection and that TNFα is required for IFNγ-mediated killing of *C. burnetii* (41, 55, 56). Reactive oxygen species are noted to have a minimal effect on controlling the bacteria, but nitric oxide (NO) species, generated from upregulation of proinflammatory cytokines including IFNγ, have been shown to inhibit the replication of *C. burnetii* by limiting the size of *Coxiella*-containing vacuoles (CCVs) (56–58). NO is not the only contributor to bacterial clearance though, as bone marrow-derived macrophages from iNOS KO mice treated with IFNγ had reduced *C. burnetii* viability despite negligible NO presence (59). Cells pretreated with IFNγ exhibit increased microbicidal activity, while cells that have already been infected are less so. Stimulating the immune system with vaccination and increasing IFNγ production with adjuvants and immunogenic antigens helps to control bacterial replication and mitigate infection. We have shown that immunizing with immunogenic antigens identified in this study results in robust IFNγ responses after antigen recall (**Figure 2**).

Adjuvants in the subunit vaccine formulations tested here include squalene oil-in-water emulsion AddaVAX, TLR4 agonist MPLA, and TLR9 agonist CpG 1018. Q-VAX elicits a skewed IgG2c/Th1 response, and these adjuvants are noted to help favor a more Th1-biased response. Formulations without adjuvant tend to be Th2-skewed (**Figures 3C, D****)**. As demonstrated in **Figure 3**, the introduction of adjuvant to a single immunogenic antigen increases proinflammatory Th1 cytokine production (IFNγ, TNFα, IL-2, and IL-6). Q-VAX exhibits a robust Th1 response as shown in **Figure 3B** and a single subunit antigen excluding the adjuvant results in a Th2-skewed response.

Immunization induces delayed type IV hypersensitivity (DTH), mediated by sensitized antigenic-specific T cells and can cause fever, malaise, and inoculation site granulomatous reactions (60). This differs from antibody-mediated hypersensitivities that may involve acute IgE antibodies (type I), IgG or IgM antibodies (type II), and immune complex formation (type III) (61). Cell-mediated inflammatory reactions may be CD4^+^- or CD8^+^-dependent and are usually limited to near the site of injection. Fratzke et al. (48) demonstrated that sensitization of C57BL/6 mice with *C. burnetii* WCV results in reactogenicity that is CD4^+^ T cell-dependent rather than CD8^+^. Sensitization can be characterized by increased production of IFNγ and IL-17a to trigger cellular immunity. Improved *C. burnetii* vaccine design aims to define the antigens necessary to elicit a protective immune response while minimizing the reactogenic DTH response. By weighing necrosis/suppuration as the biggest indicator of reactogenicity, we were able to show that five out of our six experimental vaccine formulations exhibited reduced reactogenicity when compared to positive control WCV (**Figure 4B**). Representative histological images for groups 1 and 2, the hexavalent groups, and WCV show areas of degenerate neutrophils and abscess formation, which have less of a presence in other groups (**Figure 4A**).

After demonstrating immunogenicity in mice, we moved onto the more biologically relevant guinea pig model with the top combination of protein antigens and adjuvants. We looked at dermal hypersensitivity and observed that including more antigens results in more severe reactogenicity (**Figure 4**). We observe more early-stage suppurative necrosis in the WCV group. In the group containing six antigens and IVAX-1, there is significantly more degeneration of collagen. The groups including NMI LPS developed the fewest mononuclear infiltrates when compared to WCV, indicating less reactogenicity with a reduced innate immune response (**Supplementary Figure S3B**). The increased responses when comparing groups with more antigens to those with fewer support the hypothesis that including more antigens results in more overall immunogenicity. With these data, there is crossable threshold in the number of antigens in a multivalent vaccine, and our candidates containing four antigens and fewer induce less severe reactogenic responses.

In the guinea pig challenge study, we concluded that the inclusion of more antigens and NMI *C. burnetii* LPS most closely emulates the protection from WCV (**Figure 6**). The formulation containing four antigens and NMI LPS, group 4, performs the closest to WCV with no significant difference in body weight and temperature for the entire duration of the infection. The formulation containing a single antigen with NMI LPS also performs similarly with no significant differences when compared to WCV. The groups containing all six experimental antigens exhibit protection by showing no significant difference to Q-VAX in either weight loss or temperature change (**Figures 6A, B****)**. Splenomegaly is a major indicator of *C. burnetii* infection, and the best performers in that category when compared to PBS are the two groups that contain six antigens and the group that has four antigens and NMI LPS (**Figure 6C**). It can be extrapolated from this dataset that vaccine formulations containing antigens in addition to LPS would confer better protection.

As demonstrated by our data, *C. burnetii* LPS appears to be a major player as a protective antigen. Even though it is not immunogenic in the context of our formulations and does not elicit IgG responses despite being an immunizing antigen (**Supplementary Figure S4**) and its inclusion reduces IgG production against immunizing antigens across the board, NMI LPS is still capable of conferring protection in significantly reducing splenomegaly even in an otherwise monovalent formulation, outperforming the quadrivalent formulation without LPS. The argument can be made that it is on equal standing with the hexavalent groups, as both hexavalent groups demonstrate significant differences when compared to WCV when looking at either the changes in body weight or temperature, while the monovalent group 6 does not (**Figure 6**). Although others have demonstrated immunogenicity in generating IgG against LPS (62, 63), our data show otherwise, possibly due to the nature of the AddaVAX adjuvant in forming lipid droplets and presenting emulsified antigens in certain orientations, hiding immunogenic epitopes. *C. burnetii* LPS is the only virulence factor identified in the infection of an immunocompetent animal model and has been acknowledged as a shielding molecule in allowing the pathogen to evade the host immune response (64). *C. burnetii* phase I LPS subverts host immunity by shielding the bacteria from complement and antibody binding (65). Furthermore, NMI LPS disrupts mitogen-activated protein kinase (MAPK) signaling through disruption of TLR-2 and TLR-4 and evades receptor-mediated phagocytosis by inhibiting and remodeling actin cytoskeleton organization (66–68). Addition of NMI phase I LPS as an immunogenic antigen in subunit vaccine formulations corroborates this theory and demonstrates lower signal intensities of IgG antibodies to immunizing antigens when compared to groups that did not receive the LPS (**Figures 5B, C****)**. The overall cellular response is lower in groups containing NMI phase I LPS, as there are also lower levels of Th1 cytokines (IFNγ, TNFα, IL-2) (**Figure 3**). Studies from other groups in the past have shown that patients suffering from Q fever endocarditis, a chronic manifestation of Q fever, exhibit higher levels of TNFα and IL-1β in peripheral blood mononuclear cell (PBMCs) and monocytes from blood. Introducing anti-TNF antibodies decreases uptake efficiency of *C. burnetii* into monocytes but does not play a role in intracellular killing (69).

Avirulent phase II LPS easily activates phosphorylation of MAPK p38, while exposure to phase I LPS results in no activation. P38 is one of three MAPK signaling pathway subfamilies, and phase I LPS has been shown to activate the other two pathways, c-Jun N-terminal kinase (JNK) and extracellular signal-regulated kinase (ERK) (70). This is through the presumed mechanism of phase I LPS-induced cytoskeletal remodeling, preventing colocalization of TLR-2 and TLR-4 (66). P38 is upstream of transcription factor NF-κB, which is critical for upregulating proinflammatory molecules (71, 72). Inhibition of this pathway *via C. burnetii* LPS may be the explanation for why immunogenicity readouts are lower for vaccine groups containing LPS (**Figures 3**, **6**).

*C. burnetii* NMI LPS has been studied extensively as a virulence factor and potential vaccine candidate. Studies in the past have shown that formalin-inactivated phase II *C. burnetii* lacking the LPS is a less effective vaccine and fails to protect against challenge and that LPS by itself can confer partial protection (43, 63). Mice receiving adoptive transfer of bone marrow-derived dendritic cells (BMDCs) stimulated with whole-cell protein antigen were better protected against *C. burnetii* challenge after LPS removal (73). Mimetic peptides of immunogenic epitopes on NMI LPS have also been developed and confer partial protection (62). In our studies, LPS exhibited the greatest potential in a multivalent vaccine formulation in conferring protection to guinea pigs, and further investigation is warranted to elucidate the immunogenic epitopes and the mechanisms of protection for this T cell-independent antigen. Despite its demonstrated effectiveness, the acquisition and extraction of NMI LPS are a logistical bottleneck, as the processes require government-facilitated high-containment approval, are time-consuming, and have low yield. To truly pursue LPS as a *C. burnetii* vaccine candidate antigen, alternative strategies such as peptide mimics or attenuated strains with full-length o-antigen should be considered for safety and scalable purposes.

To improve the immunogenicity of subunit vaccines, other avenues of delivery that can be considered include nanoparticle and glycoconjugate vaccines. Nanoparticles allow for the control of the surface’s structure and composition, and this quality is emulated by the AddaVAX emulsion. Nanoparticles can also be designed to encapsulate and deliver therapeutics in a controlled fashion. Some examples of current trends include lipid nanoparticles and virus-like particles (VLPs) that can be modified to incorporate adjuvants either on the surface or within in addition to candidate antigens (74, 75). This strategy offers attractive options for engineering surfaces to include multiple immunogenic antigens and epitopes, maximizing antigen presentation and cross-linking B -cell receptors to stimulate immune responses (76). Glycoconjugate vaccines are a combination of a protein to a sugar glycan, prompting multiple triggers to the immune system (77). The theme is to link B cell antigens, polysaccharides, to T -cell antigen proteins (78). Successful examples include conjugating protein to cross-reacting material (CRM) of diphtheria toxin or tetanus toxoid. Based on our own data, NMI LPS is a poor B -cell antigen in that it dampens IgG responses, but its protective qualities cannot be discounted. Future efforts looking into glycoconjugate vaccines for *C. burnetii* should explore approaches in improving the immunogenicity and detectability of LPS.

Overall, a panel of immunogenic *C. burnetii* antigens administered with potent Th1-stimulating adjuvants and native *C. burnetii* LPS demonstrates immunogenicity in mice and protection in a Hartley guinea pig aerosol model. Based on the presented data, we conclude that multivalency does result in a more protective vaccine, as we observe better performance in 4- and 6-antigen formulations. There is an upper limit for the number of antigens consequentially causing reactogenicity, with 6-antigen formulations scoring higher in necrosis and suppuration. Despite dampening immunogenic responses in both animal models, vaccines containing NMI LPS greatly improve protection. Further studies on our potential vaccine candidates need to investigate the durability in animal models to recapitulate Q-VAX’s effectiveness as a single dose conferring lifelong protection. The differences in MHC molecules between mice and humans also pose a challenge due to their structural differences, and future efforts can incorporate humanized mice to accurately represent human antigen presentation and evaluate potential T cell antigens in a preclinical setting more accurately. Another aspect to investigate is potential reactogenicity in substituting the additional two antigens present in the 6-antigen formulations vs. the 4-antigen ones. This will clarify if the reactogenicity is being caused by the addition of those specific antigens or from increasing multivalency in general. There is a balance that must be established in maximizing the number of immunogenic antigens and reducing the logistical difficulty in purifying NMI LPS that warrants further investigation. From a practical standpoint, the difficulty in protein purification serves as a major roadblock in subunit vaccine development. With the rise of mRNA technology, once immunogenic proteins are identified, we can begin to move away from the labor-intensive protein purification process and toward a scalable method of manufacturing mRNA and easily incorporating more antigens for effective multivalent vaccines.

## Data availability statement

The original contributions presented in the study are included in the article/**Supplementary Material**, further inquiries can be directed to the corresponding author/s. Data presented in the study are deposited in the Genome Expression Omnibus (https://www.ncbi.nlm.nih.gov/geo/) under the accession number GSE229293.

## Ethics statement

The animal study was reviewed and approved by UC Irvine Institutional Animal Care and Use Committee (IACUC) Animal Care and Use Review Office (ACURO) of the U.S. Army Medical Research and Materiel Command (USAMRMC).

## Author contributions

Experiments were designed by SJ, AG, DHD, and PF. SJ performed animal experiments, analyzed data, and wrote the manuscript. AG, DHD and JH-D performed animal experiments and analyzed data. AF scored histopathology slides and analyzed the data. SJ, RN, AJas, AJai, and MS printed and probed protein microarrays and SJ and DHD analyzed the data. JF formulated the vaccine formulations. LL was responsible for correspondence with funding agencies. All authors were involved in manuscript revision and editing. All authors contributed to the article and approved the submitted version.
