# Supplementary material for: Multivalent vaccines demonstrate immunogenicity and protect against Coxiella burnetii aerosol challenge
Source: Front Immunol. 2023 Jul 18;14:1192821. doi: 10.3389/fimmu.2023.1192821 (PMC10390735; doi:10.3389/fimmu.2023.1192821)
Supplement: Supplementary file 1 [file DataSheet_1.docx]

Supplementary Material

# Supplementary Figures and Tables

**Supplementary Figure 1.** **Splenocytes from mice immunized with Q-VAX generate responses to purified protein.** C57BL/6 mice (n=3) were immunized with Q-VAX intraperitoneally and their splenocytes were harvested 10 days after the prime. The pooled splenocytes were stimulated by purified protein from a downselected panel of lead antigens indicated in table 1 (“Immunogenicity” column).


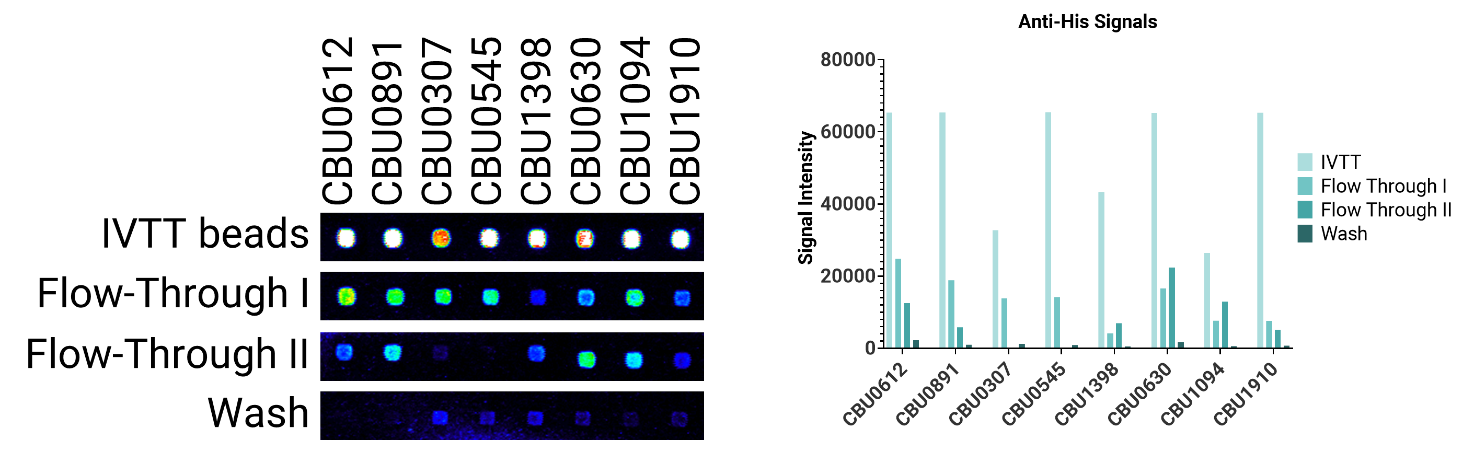


**Supplementary Figure 2.** ***Coxiella* proteins expressed in IVTT were captured on Sepharose beads for mouse immunizations.** IVTT reactions of CBU antigens were applied on GE His-TrapSpin columns. Flow-through fractions were collected and re-applied to the columns. Columns were washed to remove IVTT master mixture components. IVTT beads, flow-through, and washes were collected, printed on nitrocellulose slides, and probed against using anti-tag antibodies.


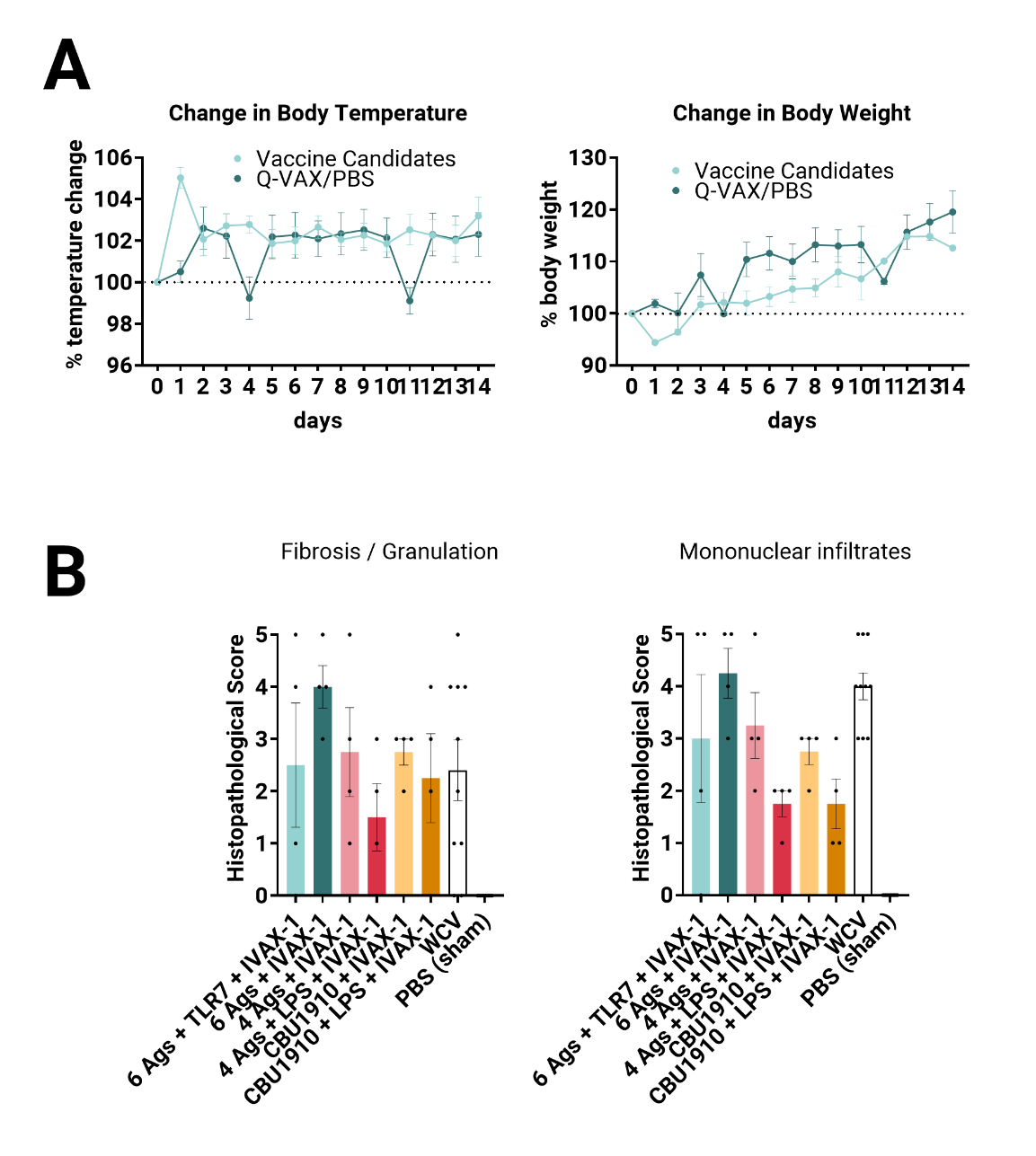


**Supplementary figure 3. Guinea pig vaccine formulations were evaluated for reactogenicity.** All animals were sensitized with Q-VAX and rested for 14 days, then intradermally administered either the 6 vaccine candidates (n=4) or Q-VAX and PBS intradermally (n=7) on shaved skin sections. **(A)** Changes in body temperature and weight in calculated percentages were recorded for 14 days after intradermal immunization. **(B)** Mean histopathological scores for experimental groups separated into different morphological categories.


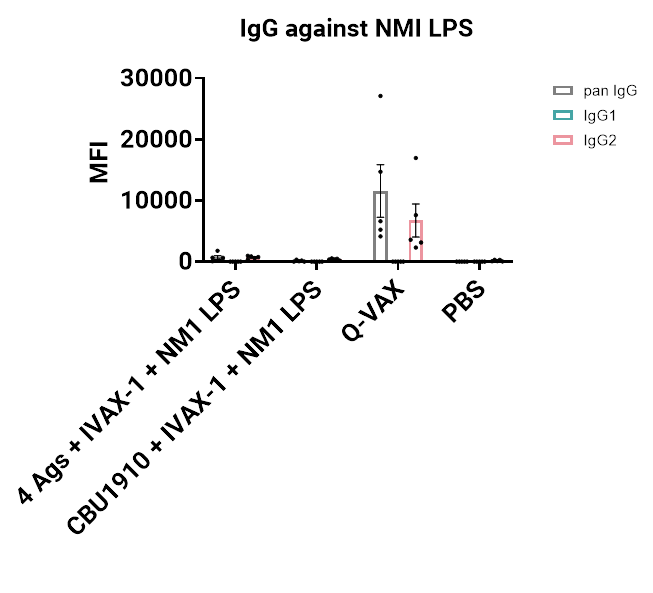


**Supplementary figure 4. IgG signal intensities against *C. burnetii* NMI LPS in vaccine formulations.** Hartley guinea pigs were primed at day 0 and boosted at day 14. Plasma from day 42 post-prime was collected and assessed on the *C. burnetii* protein microarrays for production of pan IgG, IgG1, and IgG2 against NMI LPS.
